# Can we rely on the best trial? A comparison of individual trials and systematic reviews

**DOI:** 10.1186/1471-2288-10-23

**Published:** 2010-03-18

**Authors:** Paul P Glasziou, Sasha Shepperd, Jon Brassey

**Affiliations:** 1 Centre for Evidence-Based Medicine, Department of Primary Health Care, University of Oxford, UK; 2 Department of Public Health, University of Oxford, UK; 3 TRIP, National Public Health Service, Mamhilad House, Mamhilad Park Estate, Ponytpool, UK

## Abstract

**Background:**

The ideal evidence to answer a question about the effectiveness of treatment is a systematic review. However, for many clinical questions a systematic review will not be available, or may not be up to date. One option could be to use the evidence from an individual trial to answer the question?

**Methods:**

We assessed how often (a) the estimated effect and (b) the p-value in the most precise single trial in a meta-analysis agreed with the whole meta-analysis. For a random sample of 200 completed Cochrane Reviews (January, 2005) we identified a primary outcome and extracted: the number of trials, the statistical weight of the most precise trial, the estimate and confidence interval for both the highest weighted trial and the meta-analysis overall. We calculated the p-value for the most precise trial and meta-analysis.

**Results:**

Of 200 reviews, only 132 provided a meta-analysis of 2 or more trials, with a further 35 effect estimates based on single trials. The average number of trials was 7.3, with the most precise trial contributing, on average, 51% of the statistical weight to the summary estimate from the whole meta-analysis. The estimates of effect from the most precise trial and the overall meta-analyses were highly correlated (rank correlation of 0.90).

There was an 81% agreement in statistical conclusions. Results from the most precise trial were statistically significant in 60 of the 167 evaluable reviews, with 55 of the corresponding systematic reviews also being statistically significant. The five discrepant results were not strikingly different with respect to their estimates of effect, but showed considerable statistical heterogeneity between trials in these meta-analyses. However, among the 101 cases in which the most precise trial was not statistically significant, the corresponding meta-analyses yielded 31 statistically significant results.

**Conclusions:**

Single most precise trials provided similar estimates of effects to those of the meta-analyses to which they contributed, and statistically significant results are generally in agreement. However, "negative" results were less reliable, as may be expected from single underpowered trials. For systematic reviewers we suggest that: (1) key trial(s) in a review deserve greater attention (2) systematic reviewers should check agreement of the most precise trial and the meta analysis. For clinicians using trials we suggest that when a meta-analysis is not available, a focus on the most precise trial is reasonable provided it is adequately powered.

## Background

Clinical decisions are ideally informed by a systematic review that finds, selects, and synthesizes the best primary studies that answer a single question[1]. Though the Cochrane Collaboration has completed over 3,000 systematic reviews in the past decade, this is around 15% of the number required[2], and updates are problematic. Those teaching "bedside" evidence-based medicine[3,4] suggest clinicians identify and appraise the single "largest" trial and use this as the basis of conclusions. Ideally new trials would report their results in the context of all previous relevant research, but this is rarely done[5]. This leaves practitioners wishing to base decisions on evidence with the dilemma of using a single trial or disregarding evidence.

**Figure 1:**
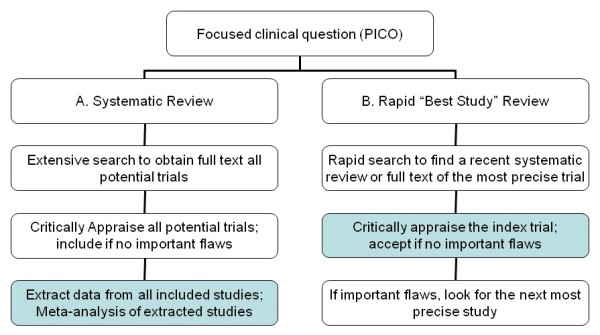
**Two options for addressing a clinical question based on research literature: (a) a full systematic review and (b) a rapid "best trial" review**. This paper compares the elements in the shaded boxes.

Doing a systematic review is not practical in daily clinical practice or for the all the questions that arise when writing a guideline. Hence if no review is currently available then clinicians or guideline writers must decide whether to rely on the best single study or invest the considerable effort involved in doing a systematic review. The workload is substantial: an analysis of 37 meta-analyses[6] showed that the average hours for a review were 1,139 (median 1110) -- or about 30 person-weeks of full-time work --with a range from 216 to 2518 hours. As a consequence, most trials have never been included in a systematic review. Clinicians therefore often attempt to do a rapid "best trial" review process which will take a few hours or less - over a hundred-fold less effort - but with clear risks of drawing inappropriate conclusions based on a limited search and single trials. The two processes are contrasted in Figure 1.

The major reasons for undertaking meta-analyses are (i) the additional statistical power provided by pooling several studies, and (ii) the ability to explore the robustness of different versions of an intervention across differing populations. However there are also downsides: pooling several small studies increases the risk of detecting publication bias rather than a real effect, and also a focus on pooling may distract from the quality of and question asked by individual studies.

Clearly a single trial can sometimes be sufficient to guide clinical decision making: for hormone replacement therapy the Women's Health Initiative (WHI) trial provides a large percentage of the available trial evidence. While systematic reviews of hormone replacement therapy may be valuable for an examination of consistency and additional outcomes, the WHI alone is a sufficient basis for many clinical decisions. However, most clinical questions will not have such a clear dominant trial. As illustrated in Figure 1, the evidence process then consists of two stages: (i) find a recent systematic review (hopefully), but if there isn't one then find the "best" trial - the most precise - and (ii) appraise and (if appropriate, and no important flaws) apply the trial results. The first step is not straightforward, but is only worthwhile if the second step will usually provide a sufficiently good answer compared with an up-to-date systematic review. Therefore, as an initial exploration of this process, we decided to examine how well and how frequently the single "best" trial might answer the clinical question.

Previous studies have looked at the special case of agreement between "mega-trials" and meta-analyses[7], but no studies appear to have examined a more representative sample of reviews. We aimed to evaluate in a random selection of systematic reviews how often, and under what circumstances, using the trials with the greatest statistical weight arrives at similar conclusions to the full meta-analysis.

## Methods

For a sample of reviews we selected the trial that contributed the most weight to the meta analysis as the most informative trial and aimed to compare its results to that of the meta analyses of the main outcome measure in the systematic review. The systematic reviews were a random sample of 200 selected from the 2,201 in issue 1, 2005 of the Cochrane Database of Systematic Reviews. For each systematic review sampled two of us (PB, SS) independently extracted data on the title, date of the last search, the number of trials included, and whether a meta-analysis was done on the trials. If a meta-analysis was undertaken, then we aimed to use the main outcome for each review. We identified the main outcome from the question in the Methods section and tried to find a match in the results. If the data was not pooled for the main outcome, then the closest match among the meta-analyzed results was chosen. We extracted data on the type of measure of effect, the point estimate and its 95% confidence interval, the p-value, and the I^2 ^for heterogeneity. For the main outcome we identified the "largest" (most precise) single trial, which we defined for this paper as the trial contributing the greatest weight to the meta-analysis. For this trial we extracted data on the weight and size of the trial, the point estimate and 95% confidence interval, and p-value. We also recorded the direction of outcome of all of the trials (in favor of, neutral, or against the main treatment). We relied on the authors of the review to choose whether a fixed or a random effects model was appropriate, and did not redo their meta-analytic results.

### Analyses

We compared the estimates of effect from the trial with the most weight with that from the whole meta-analysis. To use consistent methods within and between reviews, we calculated the p value for the trial by a z-test (log-transformed for dichotomous data) rather than use the p-values calculated by various means in the individual trials.. We compared the p-value of the trial with the p-value obtained from the meta-analysis. To dichotomize the p-value results, we classed trials and meta-analyses as statistically significant if the p-value was less than 0.05.

## Results

Of the 200 randomly selected Cochrane reviews, 132 (66%) had a meta-analysis which included 2 or more trials. Of the other 68 reviews, 12 had no trials, 35 included only a single trial, and 21 had no meta-analysis as the trials could not be pooled, Of those trials with a meta-analysis, the average number of trials included in the meta-analysis of the main outcome was 7.3 trials, which is similar to a previous analysis of all Cochrane reviews in 2001[8]. The median weight of the most precise trial within the meta-analysis was 51%, that is, the majority of the weight of the meta-analysis. The majority had little heterogeneity with a median I^2 ^of 1.2%. Further details are given in Table 1.

**Table 1:** Components of the 132 systematic reviews that included a meta-analysis.

	Mean (SD)	Median	Range	Comments
Trials in the meta analysis	7.3 (11.6)	4.0	2 to 184	Does not include the 35 reviews with a single trial

Weight of the most precise trial	50% (22.0)+-	51%	3.3 to 96.4	Weight is based on the inverse variance calculated in RevMan

Sample size in most precise trial	1148 (7392)	183	14 to 82,892	

P value for meta analysis	0.21 (0.29)	0.03	0.0 to 0.99	

Degree of Heterogeneity - I^2^	26.6% (30%)	1.2%	0% to 90%	Does not include the 35 reviews with a single trial

For 184 relevant reviews (for 12 reviews there were no trials, and in 4 without meta-analysis we could not tell which was the trial with the greatest weight) the trial with the most weight was present in MEDLINE in 89% (162 of 184) of the reviews. 20 were only found in other databases, and 1 was unpublished.

**Figure 2:**
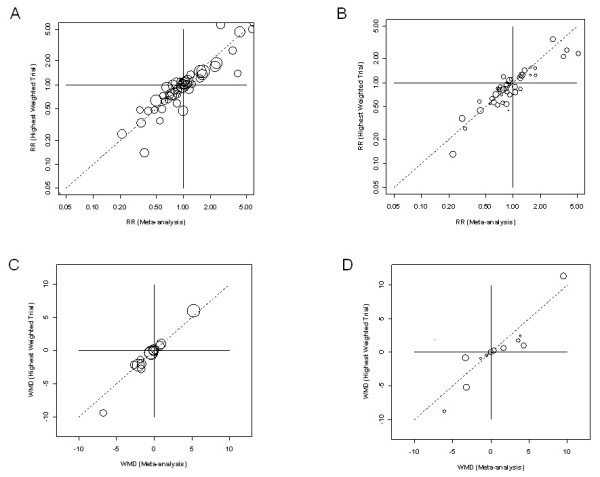
**Estimates of effect for the index (highest weighted) trials compared to the meta-analysis (larger circles have higher weight within the review)**: A. Relative Risks (RR) where the index trial is more than 50% of the weight (A1) and less than 50% (A2); B. Weighted Mean Differences where the index trial is more than 50% of the weight (B1) and less than 50% (B2) - note: for the 4 trials and meta-analyses with WMDs more than 10 (or -10) we rescaled to fit.

The estimates of effect for the most precise trial and meta-analysis were generally similar. The Pearson rank correlation was 0.90. Figure 2 is a scatter plot of the results (excluding those meta-analyses that had only a single trial, which would all lie along the diagonal), subgrouped by whether the index trial was more or less than 50% of the weight in the meta-analysis, irrespective of the absolute size of the trial. The upper figures are for ratio measures (relative risk, odds ratio, or hazard ratio); the lower figures are for weighted mean differences. The two off diagonal quadrants show some disagreements in direction of effect, but mostly when both estimates are close to an RR of 1.

**Figure 3:**
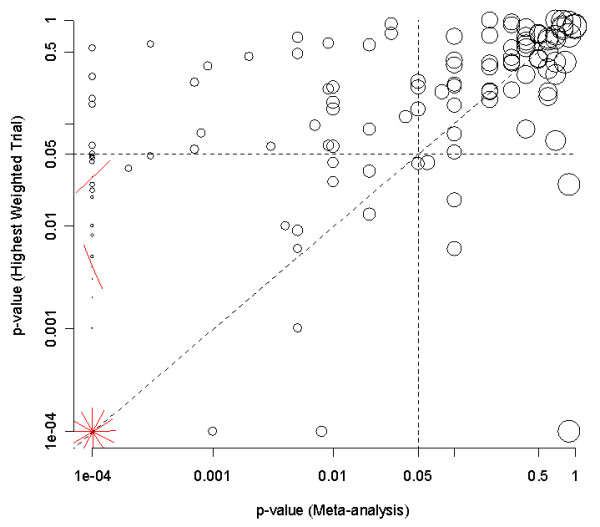
**Scatter plot of p-values from the 132 meta-analyses (x-axis) compared to p-values from the most precise trial (y-axis)**. Values in the lower left and upper right quadrants are in agreement; those in (i) the upper left and (ii) lower right are in disagreement.

Of the 167 with either a summary result, a meta-analysis, or a single trial 86 (51%) showed statistically significant results. Figure 3 shows the comparison of p-values from the most precise trial and the meta-analysis. The dotted lines divide the plot into a 2 × 2 table with the two boxes along the diagonal representing areas of "agreement" and the two off diagonal areas (between the dotted lines and axes) representing the "disagreements".

These results are summarized in Table 2, which shows that the statistical conclusions agreed in 135 pairs (81%). Most disagreements were where the systematic review found a statistically significant result when the most precise single trial did not. However, in 5 cases, the most precise single trial was significant when the meta-analysis was not on the same outcome measure. These disagreements were surprising, and hence we sought to understand the possible reasons in more detail.

**Table 2:** Agreement on statistical significance between the overall results of the systematic review and the most precise single trial.^1^

	Meta-analysis or single trial	
**Large Trial**	**P < 0.05**	**P > 0.05**

P < 0.05	55 (including 14 single trial reviews)	5

p > 0.05	31	70 (including 15 single trial reviews)

^1 ^Six single trial reviews are excluded due to insufficient data

### Why do they disagree? I - SR non-significant but big trial significant

The 5 disagreements are detailed in Table 3. In one case (row 1 of Table 3) the disagreement was minor (p = 0.06 versus 0.04), but in the other 4 cases the disagreement was more substantial. However, in no case was the direction of effect reversed. Of note is the high degree of heterogeneity found in all 5 cases, with the least being an I^2 ^of 54%. In two cases there was also important clinical heterogeneity, so that the intervention in the most precise trial appeared to be importantly different to the intervention in other trials. For example, in the review of several trials of DOTS (Directly Observed Therapy - having a health care worked observe the patient take their daily medication) for tuberculosis, the location of the intervention differed from patients needing to go for daily visits to a health centre to home visits by a health care worker or family member, but there are also methodological concerns with the trial that might explain the difference. The latter is clearly easier for patients to adhere to, and showed a positive effect whereas the pooled results did not. The 2 trials in infantile spasm used very difference dosages, and again meta-analytic pooling is questionable.

**Table 3:** Reviews where the most precise trial was statistically significant but the meta-analysis was not, with the p-value and effect estimates* of both.

Title of review	Outcome measure	Number of trials	P value of meta analysis vs most precise trial *(trial weight)*	Estimate of effect for meta analysis vs most precise trial	**I**^**2**^
Prophylactic antibiotics for preventing early central venous catheter Gram positive infections in oncology patients	Catheter related sepsis	4	0.06 vs <0.04 *(51%)*	OR 0.55 vs 0.35	54%

Azithromycin for acute lower respiratory tract infections	Clinical failure	14	0.9 vs < 0.001 *(14%)*	RR 0.96 vs 0.52	58%

Directly observed therapy for treating tuberculosis	Cure	4	0.1 vs < 0.006 *(60%)*	1.06 vs 1.13	64%

Laparoscopic colposuspension for urinary incontinence in women	Number subjectively cured within 18 months	3	0.9 vs < 0.025 *(56%)*	RR 1.0 vs 1.05	73%

Treatment of infantile spasms	Cessation of spasms	2	0.1 vs < 0.02 *(65%)*	OR 2.57 vs 1.35	69%

* OR is the Odds Ratio; RR is the Relative Risk; I^2 ^is the heterogeneity proportion

### Why do they disagree? II - SR significant big trial non-significant

In 31 of 86 statistically significant meta-analyses the most precise trial was not statistically significant. This "disagreement" is less surprising, as many of these most precise trials were still clearly underpowered for the key outcome measure. Of note is that the trial confidence interval did include the systematic review's overall estimate in 25 of the 31 cases, and the confidence intervals overlapped in all but 1 case.

## Discussion

Among the reviews we examined the level of statistical significance between the majority of meta analyses and the trials with the most weight was in agreement. In particular if the trial with the most weight is statistically significant, then the meta-analysis is usually also statistically significant. However, if the trial with the most weight is non-significant, the meta-analysis was statistically significant in about 1/3 of the reviews (31/101, 31%), suggesting that even those trials with the most weight were often underpowered.

A limitation of our analysis is that we have treated meta-analysis as if it were a "gold standard". However there are a number of studies suggesting they are not. For example, in an analysis of multiple systematic reviews on 17 topics in complementary medicine[9] the authors stated "we were surprised by the number and scale of the discrepancies." Among reviews comparing the results of large-trials (trials with more than 1,000 patients) with the results of meta-analyses agreement ranging from kappas of 0.22 to 0.72 was reported, depending on the methods used. One of the better analysis [7] observed that large trials disagreed with meta-analyses 10% to 23% of the time. These analyses treated the large-trial as the gold standard, but again large trials of the same clinical question do not always agree. A study of 422 pairs of mega-trials found only modest agreement in the results with a weighted kappa of 0.40, though only 2% of trial pairs showed a statistically significant differences in opposite directions[10].

As indicated in the introduction, we have not addressed how to find the "best" trial, but only the next step of what we could conclude if we did. However identifying the best or most precise trial may not be simple, and further research is warranted to examine different methods to identify key trials among all the trials. These are more likely to be in MEDLINE, be in major journals, and be multi-center, but we don't know the accuracy of different filtering heuristics. Filters or tagging for key trials would be valuable when no systematic review is available. Since the power of studies is not always proportional to the sample, the "most precise" trial may be difficult to identify even if it is among the search results. The closest equivalent would be to base the choice on the width of confidence intervals, but these are often not reported in the abstracts. A potential alternative would be the number of events[11], but again this is often not reported in abstracts. Clearly if rapid searching is to be viable, more research is needed into these options. Finally as suggested in Figure 1, even if the most precise trial is identified, it still needs to be critically appraised, and, if unacceptably flawed, the second most precise trial examined, etc.

For clinicians and guideline authors trying to use systematic reviews and trials, we cautiously suggest that when a meta-analysis is not available, then a focus on the most precise trial is reasonable provided it is adequately powered (that is the confidence intervals exclude values that would change the clinical decision) and adequately conducted. If there is no existing systematic review, those needing an answer to a clinical question, but without the time or resources to undertake a meta-analysis, might search for the most precise well-conducted trial and carefully check whether the study was sufficiently large. However, particular caution is needed about making negative conclusions based on small trials.

Though not our primary focus, the results also have some implications for systematic reviewers. The key trial(s) included in a review may deserve greater attention. If a trial provides a substantial portion of the weight in a review, it deserves careful analysis and comparison with the other trials. This is in part an error check: the index trial will usually be close the overall result. Such a check may have prevented the error that occurred in one Cochrane review where the direction of effect of most trials had been coded in the wrong direction[12]. The opposite conclusion from the index trial could have alerted the reviewers to a possible problem.

It may be prudent for systematic reviewers to check agreement of the most precise trial and the meta analysis. If the results of the large trial and the meta analysis are inconsistent (as in Table 3) then reviewers should be careful about pooling for two reasons. First, the smaller trials may be of lower methodological quality: an analysis of 190 trials within 14 meta-analyses, smaller trial were 3 times more likely to be of low quality[13].

## Conclusions

In general, a systematic review is preferred particularly when all trials are underpowered. Even when a dominant well-powered study or trials exist other trials can still provide additional useful information, e.g, subgroups, robustness of findings across groups. Hence systematic reviews are generally likely to be among the most cost-effective types of research[14]. However, given the current limited resources to conduct systematic reviews, the full process might be reserved for crucial issues or prior to large trials, and, whether or not a meta-analysis is done, greater attention should be given to key trials. Further research is warranted on methods for efficiently finding larger trials.

## Competing interests

The authors declare that they have no competing interests.

## Authors' contributions

PG designed the study; JB and SS analysed the 200 systematic reviews and extracted data; all authors contributed to the analysis and writing.

## Pre-publication history

The pre-publication history for this paper can be accessed here:

http://www.biomedcentral.com/1471-2288/10/23/prepub
